# Surgical Intervention in Pediatric Marfan Syndrome: A Multiinstitutional Study

**DOI:** 10.1177/21501351251335474

**Published:** 2025-05-15

**Authors:** Mario O’Connor, Andrew Well, Maria E. Hoyos, Catherine E. Stauber, Charles D. Fraser, Hugo Martinez

**Affiliations:** 1 Texas Center for Pediatric and Congenital Heart Disease, 377659 The University of Texas at Austin Dell Medical School, Austin, TX, USA; 2 Pediatric Cardiac Surgery, 12328 Vanderbilt University Medical Center, Nashville, TN, USA

**Keywords:** Marfan syndrome, congenital heart disease, outcomes, long-term outcomes

## Abstract

**Background:**

Individuals with Marfan syndrome (MFS) are at risk for the development of cardiovascular complications. Although the majority of MFS patients do not require cardiac surgery until adulthood, cardiovascular disease in the pediatric MFS population is still notable.

**Methods:**

This is a retrospective of the Pediatric Health Information System from January 2004 to December 2023. All patients <18 years of age with a diagnosis of MFS who underwent aortic (Ao) and/or mitral valve (MV) surgery were included.

**Results:**

A total of 343 patients were identified, with 115 (34%) females, 190 (55%) White non-Hispanic, and a median age of 13.0 [interquartile range (IQR): 9.0–16.0] years at surgery. Among the cohort, 241/343 (70%) underwent an aortic procedure, 40/343 (12%) aortic + MV procedure, and 62/343 (18%) MV procedure. Median hospital length of stay was 6.0 [IQR: 5.0–9.5] days, 9/343 (3%) patients required extracorporeal membrane oxygenation, and 4/343 (1%) died in-hospital. Freedom from any reintervention at 15 years was 48.8% (95% CI: 34.0–70.0). Multivariable Cox regression analysis revealed that older age at the time of surgery was associated with a reduced risk of reintervention (hazard ratio: 0.94; 95% CI: 0.89–0.99, *p* = 0.023). The overall survival rate at 15 years was 94.2% (95% CI: 90.5–98.0), and no factors were associated with increased mortality.

**Conclusions:**

Operations for cardiovascular complications of MFS can be performed safely in appropriately selected children with low morbidity and mortality and with good short-term and long-term outcomes. These findings support the safety and efficacy of surgical intervention in this population.

## Introduction

Marfan syndrome (MFS) is a genetic disorder most commonly caused by mutations in the FBN1 gene.^1,2^ It represents the most common heritable connective tissue disorder. It is characterized by ocular, skeletal, and cardiovascular abnormalities. Cardiovascular abnormalities include valvular insufficiency, aortic aneurysms, and aortic dissections.^3,4^ If left untreated, cardiovascular abnormalities cause death in one-half of all patients within the first 4 decades of life.^5^

Although MFS typically manifests in adulthood, pediatric patients can develop critical cardiovascular complications necessitating early surgical interventions.^6,7^ Surgical management advancements have significantly improved outcomes in these patients.^8^ However, the risk of aortic dilation, rupture, and heart failure is significant in MFS patients, and interventions such as aortic surgery, valve replacement, and mitral valve (MV) repair have become key components in management, with data demonstrating that surgical intervention prolongs survival in patients with MFS.^5^

Previous studies have highlighted the safety, efficacy, and importance of timely surgical intervention to prevent life-threatening complications in the young adult and adult populations.^9,10^ However, there is limited data on pediatric patients, and outcomes remain limited to single-center studies.^5,11,12^ While an effort using a larger cohort was attempted, long-term outcomes of patients who underwent cardiovascular surgery during childhood were limited.^13^

This study focuses on describing surgical interventions, and short- and long-term outcomes in a cohort of pediatric MFS patients undergoing cardiovascular surgery using a nationwide database.

## Patients and Methods

### Data Source

This study is a retrospective analysis utilizing the Pediatric Health Information System (PHIS) database, spanning from January 1, 2004 to December 30, 2023. Managed by the Children's Hospital Association, PHIS contains administrative and billing data from 49 pediatric hospitals, representing approximately 20% of pediatric hospitalizations across the United States.^14^

The data submitted to PHIS is anonymized at the point of entry and subjected to comprehensive quality assurance measures. Each hospital visit is assigned a distinct identifier, allowing for the longitudinal tracking of patients within individual facilities, although tracking across different hospitals is not possible. PHIS data include the primary diagnosis, up to 41 additional diagnoses, the principal procedure, and as many as 41 supplementary procedures.

### Study Population

This study included all pediatric patients with an MFS diagnosis who underwent cardiovascular surgical interventions at <18 years of age. Surgical procedures were grouped into 3 main groups: aortic procedures, aortic + MV procedures, and MV procedures. Aortic procedures included all patients who underwent aortic valve (AoV) interventions, as it is likely that all these patients underwent a root intervention during the same operation and were not coded appropriately. To analyze trends in surgical practices and patient outcomes over time, cases were further stratified into three distinct time frames: 2004–2009, 2010–2015, and 2016–2023. Additionally, age was stratified into 4 groups: <1 year, 1–7 years, 8–15 years, and >15 years, to describe the incidence of procedures by age. Accurate identification of diseases and procedures was ensured using validated International Classification of Diseases (ICD) codes.^13^

### Study Outcomes

The primary outcomes of interest were to describe indications for surgery and perioperative survival. Secondary outcomes included, surgical complications, freedom from surgical reinterventions, and overall survival probability. The surgical complications assessed were acute renal failure, pericardial effusion, and complete atrioventricular (AV) block.

Pericardial effusion was defined as any patient with a diagnosis of pericardial effusion and need of intervention, such as pericardiocentesis. Complete AV heart block was defined as any patient with a diagnosis of complete AV block and placement of a pacemaker after a surgical procedure.

### Statistical Analysis

Descriptive statistics were reported for demographics, clinical characteristics, and outcomes. Categorical variables are reported as *n* (%). Length of stay (LOS) is reported in median [interquartile range (IQR)] days. Chi-square and Fisher's exact test were utilized to analyze noncontinuous variables. Kruskal–Wallis test was utilized to analyze the LOS comparison between groups. Kaplan–Meier curves, univariate, and multivariable Cox regression analyses were utilized to assess long-term outcomes. Statistical analyses were performed using R and RStudio.^15^

## Results

### Study Population and Demographics

From January 1, 2004, to December 30, 2023, 343 patients with MFS underwent cardiovascular surgery. Among them, 115 (34%) were female, and the median age at surgery was 13.0 [IQR: 9.0–16.0] years. The majority of procedures (*n* = 155/343, 45%) occurred in the most recent era (2016–2023) (Table 1).

**Table 1.** Demographics.

Variable	Overall, *n* = 343	Aortic procedure, *n* = 241 (70)	Aortic + MV procedure, *n* = 40 (12)	MV procedure, *n* = 62 (18)
Age (median) in years [IQR]	13.0 [9.0–16.0]	14.0 [12.0–16.0]	11.0 [7.7–15.2]	8.0 [2.5–13.7]
Gender				
Female	115 (34)	64 (27)	18 (45)	33 (53)
Race				
White non-Hispanic	190 (55)	133 (55)	22 (55)	35 (56)
Hispanic	59 (17)	43 (18)	7 (18)	9 (15)
Black	51 (15)	41 (17)	4 (10)	6 (10)
Other	43 (13)	24 (10)	7 (18)	12 (19)
Era				
2004–2009	83 (24)	44 (18)	15 (38)	24 (39)
2010–2015	105 (31)	68 (28)	19 (48)	18 (29)
2016–2023	155 (45)	129 (54)	6 (15)	20 (32)

Abbreviations: AoV, aortic valve; IQR, interquartile range; MV, mitral valve.

### Cohort Outcomes

**Figure 1.**
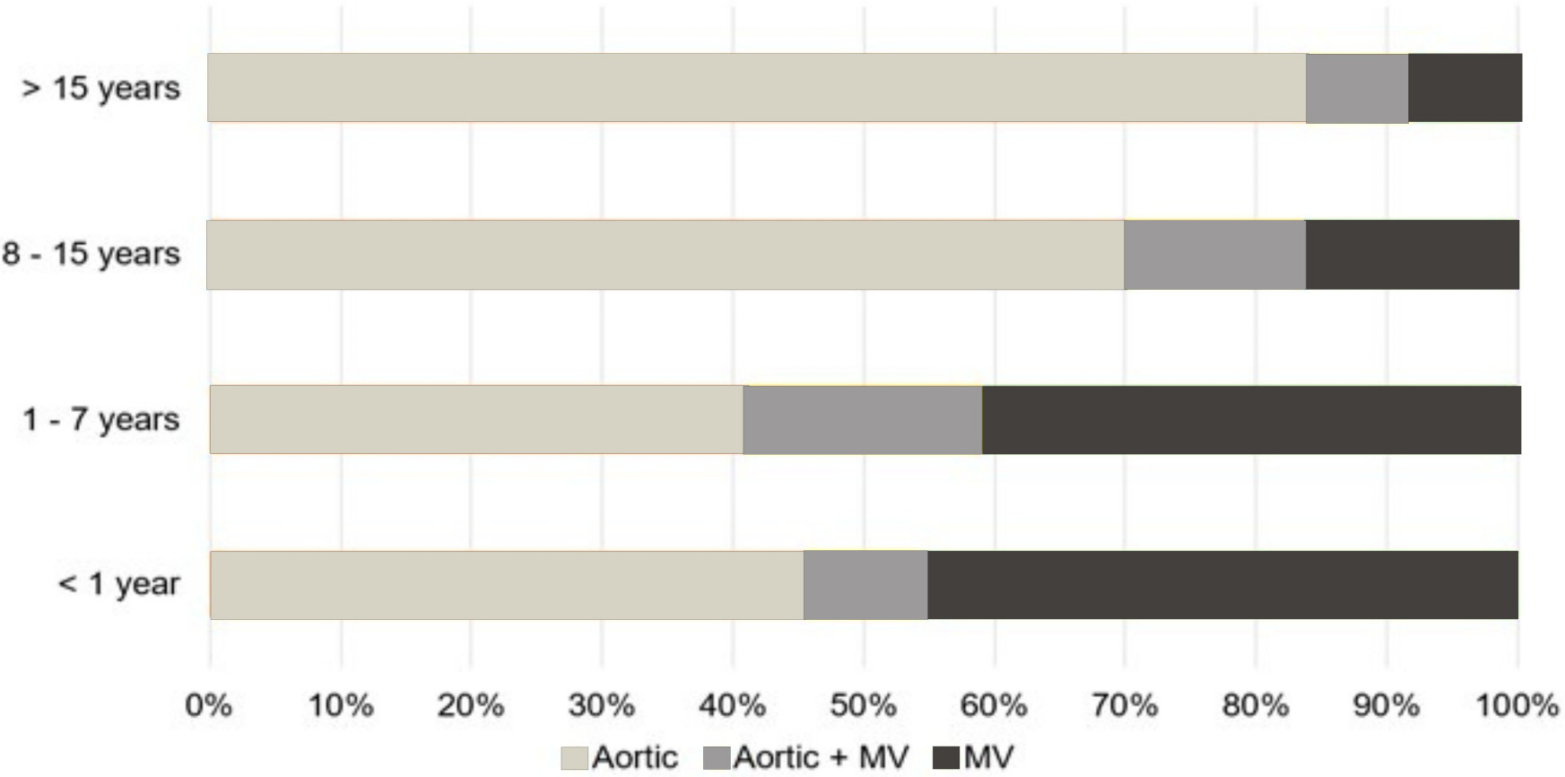
Procedure Distribution by Age.

Overall, the most common procedures performed were aortic procedures (*n* = 241/343, 70%; Table 1). In patients <1 year of age (*n* = 22), MV (*n* = 10/22, 45%) and aortic procedures (*n* = 10/22, 45%) were the most common. Among patients aged 1–7 years (*n* = 44), MV (*n* = 18/44, 41%) (and aortic procedures (*n* = 18/44, 41%) were the most common. In patients aged 8–15 years (*n* = 140), aortic procedures (*n* = 98/140, 70%) were the most common. Finally, in patients >15 years of age (*n* = 137) , aortic procedures were the most frequently performed (*n* = 115/137, 84%) (Figure 1). A total of 260/343 (76%) patients underwent valve procedures. For those patients who underwent a valve procedure, replacements were done in half of the cases (*n* = 129/260, 50%; Supplemental Table 1).

The median LOS for the entire cohort was 6.0 [IQR: 5.0–9.5] days. Nine of 343 (3%) patients required extracorporeal membrane oxygenation (ECMO). Out of a total of 343 patients 11 (3%) had a pericardial effusion, 19 (6%) had acute renal failure, and 5 (2%) had complete AV block (Table 2).

**Table 2.** Outcomes and Complications.

Procedure	Overall, *n* = 343	Aortic procedure, *n* = 241 (70)	Aortic + MV procedure, *n* = 40 (12)	MV procedure, *n* = 62 (18)
Median length of stay (days) [IQR]	6.0 [5.0–9.50]	6.0 [5.0–8.0]	8.0 [6.0–12.2]	6.0 [5.0–12.7]
Median preoperative length of stay (days) [IQR]	0.0 [0.0–0.0]	0.0 [0.0–0.0]	0.0 [0.0–0.0]	0.0 [0.0–1.0
Median postoperative length of stay (days) [IQR]	6.0 [5.0–9.0]	6.0 [5.0–8.0]	8.0 [5.7–12.2]	6.0 [4.2–11.0]
ECMO	9 (3)	5 (2)	1 (3)	3 (5)
Mortality	4 (1)	2 (1)	1 (3)	1 (2)
Complications				
Pericardial effusion	11 (3)	3 (1)	5 (13)	3 (5)
Acute renal failure	19 (6)	13 (5)	1 (3)	5 (8)
Complete AV block	5 (2)	0	2 (8)	3 (5)

Abbreviations: AoV, aortic valve; ECMO: extracorporeal membrane oxygenation; IQR, interquartile range; MV, mitral valve.

**Figure 2.**
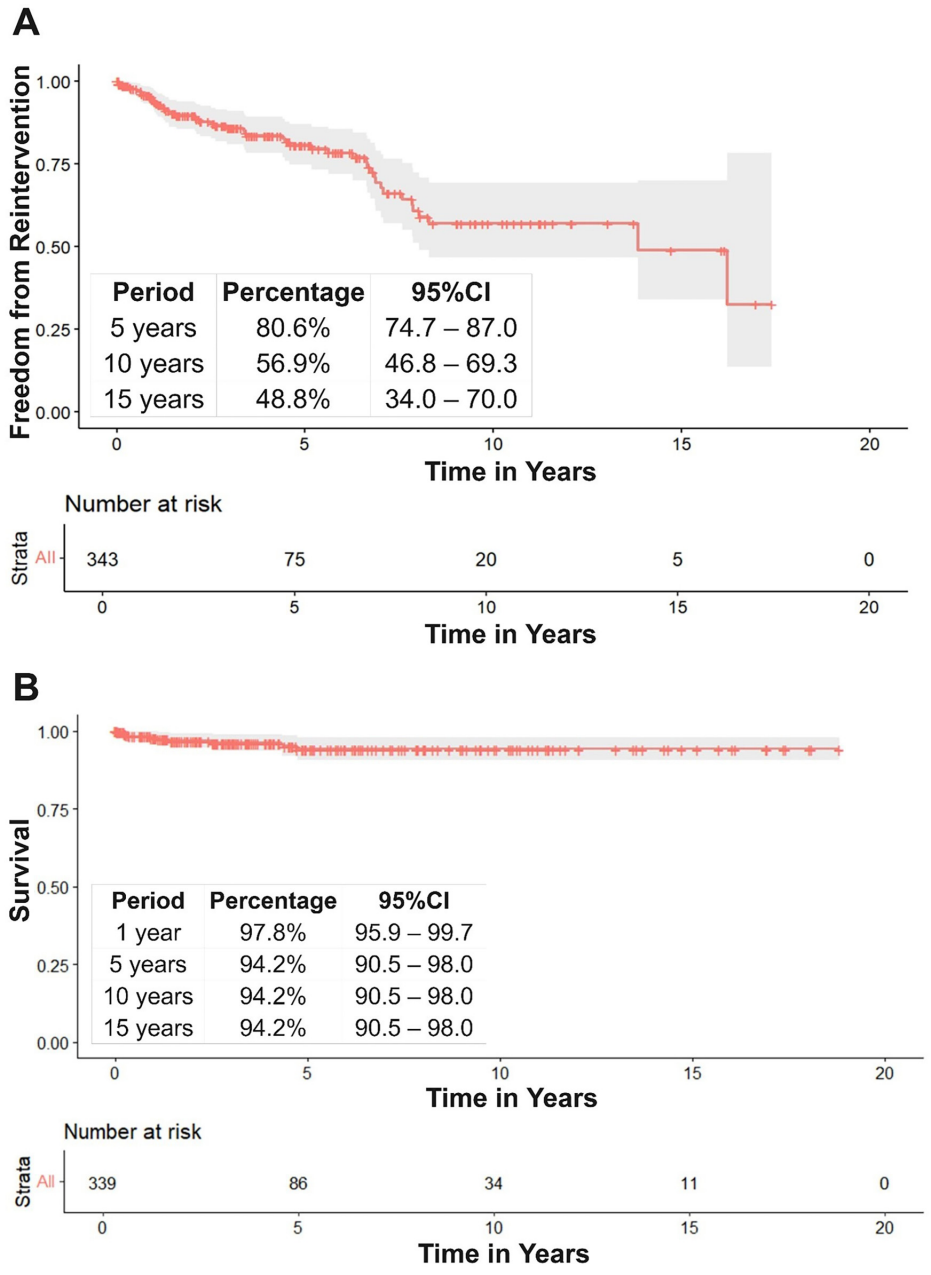
Freedom from Reintervention and Survival.

Over a median follow-up time of 1.6 [IQR: 0.05–5.03] years, 51/343 (15%) patients required a cardiac reintervention. Aortic procedures were the most common reason for reintervention (*n* = 36, 71%) (Table 3). Freedom from reintervention at 15 years was 48.8% (95% CI: 34.0–70.0) (Figure 2A), and the overall survival rate was 94.2% (95% CI: 90.5–98.0) (Figure 2B). In multivariable Cox regression analysis, younger age at surgery was associated with increased risk for any cardiac reintervention (hazard ratio: 0.94; 95% CI: 0.90–0.99, *p* = 0.023; Supplemental Table 2)

**Table 3.** Reinterventions.

Procedure	Overall, *n* = 343	Aortic procedure, *n* = 241 (70%)	Aortic + MV procedure, *n* = 40 (12%)	MV procedure, *n* = 62 (18%)
Total number of patients	51 (15%)	28 (12%)	8 (20%)	15 (24%)
Aortic/aortic + AoV/AoV procedure	34 (67)	22 (79)	5 (63)	7 (47)
Aortic	5 (15)	4 (18)	—	1 (14)
AoV replacement	23 (68)	14 (64)	5 (100)	4 (57)
AoV repair	6 (18)	4 (18)	—	2 (29)
Aortic + mitral	6 (12)	2 (7)	2 (25)	2 (13)
AoV replacement	3 (50)	1 (50)	1 (50)	1 (50)
AoV repair	2 (33)	1 (50)	1 (50)	—
MV repair	3 (50)	1 (50)	—	2 (100)
MV replacement	3 (50)	1 (50)	2 (100)	—
MV procedure	10 (20)	3 (11)	1 (13)	6 (40)
Replacement	6 (60)	2 (67)	1 (100)	3 (50)
Repair	4 (40)	1 (33)	—	3 (50)
				
RV to PA conduit	1 (2)	1 (4)	—	—

Abbreviations: AoV, aortic valve; MV, mitral valve; PA, pulmonary artery; RV, right ventricle.

### Aortic Procedure Cohort

Of the total cohort, 241/343 (70%) patients underwent an aortic procedure. Among these, 64/241 (27%) were female, 133/241 (55%) were White non-Hispanic, and the median age at surgery was 14.0 [IQR: 12.0–16.0] years (Table 1). Of note, 6/241 (2%) patients had a Ross procedure. The median LOS was 6.0 [IQR: 5.0–8.0] days. Postoperatively, 5/241 (2%) patients required ECMO support, 3/241 (1%) had pericardial effusion, and 13/241 (5%) had acute renal failure. There were 2/241 (1%) in-hospital mortalities reported in this group (Table 2).

During the study period, 28/241 (12%) patients underwent at least 1 reintervention. Among these, 22/28 (79%) underwent an aortic procedure, 2/28 (7%) underwent an aortic +  MV procedure, 1/2 (50%) underwent MV replacement, and 3/28 (11%) underwent an MV procedure, with 2/3 (67%) undergoing MV replacement. Freedom from any reintervention in this cohort was 93.4% (95% CI: 89.5%–97.5%) at 1 year, 84.1% (95% CI: 77.4%–91.4%) at 5 years, and 61.3% (95% CI: 48.1–78.0) at 15 years.

When looking at only the 6 patients who underwent a Ross procedure, 50% (*n* = 3) were performed in the modern era (2016–2023) and the median LOS was 47.5 [40.5–69.5] days. Postoperatively 1/6 (17%) patient required ECMO and no mortalities were reported in this group. Over a median follow-up time of 3.8 [IQR: 0.6–11.6] years, 1/6 (17%) patient underwent reintervention in the right ventricle–pulmonary artery conduit, and no reinterventions in the AoV or pulmonary valve were seen. Freedom from any reintervention at 1 year was 100% (95% CI: 100–100) and 50% (95% CI: 12.5–35.4) at 8 years (Supplemental Figure 1A).

### Aortic Valve and Mitral Valve Procedure Cohort

A total of 40/343 (12%) patients underwent aortic + MV procedures. Among these, 18/40 (45%) were female, 22/40 (55%) were White non-Hispanic, and the median age at surgery was 11.0 [IQR: 7.7–15.2] years (Table 1). Of these patients, 35/40 (88%) underwent MV repair (Supplemental Table 1). The median LOS was 8.0 [IQR: 6.0–12.2] days. Postoperatively, 1/40 (3%) patient required ECMO, 5/40 (13%) pericardial effusion, 1/40 (3%) acute renal failure, and 2/40 (5%) complete AV block. In this subgroup 1/40 (3%) in-hospital mortality was reported.

During the study period, 8/40 (20%) patients required at least one reintervention. Of these, 5/8 (63%) underwent an aortic procedure, 2/8 (25%) underwent an aortic +  MV procedure, both of whom required MV replacement, and 1/8 (13%) underwent an MV procedure, which was a replacement. Freedom from any reintervention in this cohort was 96.2% (95% CI: 89.0%–100%) at 1 year, 64.3% (95% CI: 45.4%–91.1%) at 5 years, and 51.4% (95% CI: 29.4–90.1) at 10 years (Supplemental Figure 1B).

### Mitral Valve Procedure Cohort

Of the total cohort, 62/343 (18%) underwent the MV procedure. Among these, 33/62 (53%) were female, 35/62 (56%) were White non-Hispanic, and the median age at surgery was 8.0 [IQR: 2.5–13.7] years (Table 1). Of these patients, 24/62 (39%) had MV replacement, and 38/62 (61%) had MV repair (Supplemental Table 1). The median LOS was 6.0 [IQR: 5.0–12.7] days. Postoperatively, 3/62 (5%) patients required ECMO, 3/62 (5%) had pericardial effusion, 5/62 (8%) had acute renal failure, and 3/62 (5%) had complete AV block. There was 1/62 (2%) in-hospital mortality reported in this group (Table 2).

During the study period, 15/62(24%) patients in this group required at least 1 reintervention. Among these, 7/15 patients (47%) underwent an aortic procedure, 6/15 (40%) patients underwent an MV procedure, 3/6 (50%) underwent MV replacement, 3/6 (50%) underwent MV repair, and 2/15 (13%) patients underwent aortic + MV procedure, with both of the patients undergoing MV repair (Table 3). Freedom from any reintervention in this cohort was 93.2% (95% CI: 86.0%–100.0%) at 1 year, 80.1% (95% CI: 68.6%–93.6%) at 5 years, and 33.5.8% (95% CI: 13.6%–82.5%) at 15 years (Supplemental Figure 1C).

## Discussion

This study analyzes cardiovascular surgery in pediatric patients with MFS over a period of 19 years using a multiinstitutional database, demonstrating low morbidity and mortality. Overall, the most common procedures performed were aortic procedures accounting for 70% (241/343) of the surgeries, followed by MV procedures (62/343, 18%) and aortic + MV procedures (40/343, 12%). Valve replacements were more common than valve repairs. Postoperative complications occurred in 154/343 (45% of patients, with pleural effusion the most prevalent. The in-hospital mortality rate was 4/343 (1%) and median LOS was 6 [IQR: 5–9] days. Younger age at the time of surgery was associated with a higher likelihood of reintervention, with a freedom from reintervention at 15 years of 48.8% and an overall survival rate of 94.2%.

Marfan syndrome in the pediatric population presents due to its early onset of cardiovascular manifestations.^6,16^ Unlike adults, pediatric patients face a longer risk period for complications such as aortic dilation, valve dysfunction, and the need for reinterventions from growth and disease progression.^17,18^ There is limited information available to guide clinical decisions, as many of the current studies focus on adult populations or are single-center.^6^ Standardized care and long-term management strategies for pediatric patients are lacking. Understanding the short- and long-term outcomes of pediatric MFS patients undergoing cardiovascular surgery is critical to addressing these challenges, optimizing care, and providing effective counseling for families.

Previous studies have highlighted the impact of gender on MFS phenotype and the need for surgical intervention. In our study, 228/343 (66%) patients were male and, in every procedure, the majority were male, except for the MV procedure group, where 53% (33/62) were female. Roman et al^19^ reported that male adult patients were more likely to present with aortic dilation, and aortic regurgitation and more likely to require surgical intervention. Our data match these findings with male pediatric patients showing higher intervention rates. The influence of gender on disease severity and progression is unknown; however, it has been hypothesized to be related to fibrillin gene expression.^20^ Future research should focus on the impact of gender on the disease prognosis, progression, and severity during childhood.

That being said, it is worth emphasizing the complexity of cases performed during the study period, with 111/343 (32%) patients undergoing combined procedures and 5/343 (1%) having 3 procedures performed. Addressing coexisting pathologies during a single operation in MFS patients has been well documented in the adult population, with studies demonstrating favorable short- and long-term outcomes.^21–23^ Although our study includes a relatively small number of cases, it is in line with these findings, showing that combined procedures are associated with good short-term and long-term outcomes. This highlights the effectiveness and safety of addressing multiple pathologies in a single intervention within the pediatric population; however, studies with a larger sample size with clinical data are needed to better understand the outcomes.

In this study, 260/343 (76%) patients underwent a valve procedure. Among those requiring an AoV procedure, AoV replacement was more commonly performed. While the literature on AoV repair in adult MFS patients demonstrates that AoV repair is safe, feasible, and associated with good long-term outcomes, there is a lack of comparable data in pediatric populations.^24–26^ Unfortunately, the data used in this study does not provide information on valve morphology or functionality, limiting our ability to understand the rationale behind choosing valve replacement over repair. This warrants further investigation to understand factors associated with an increased risk for AoV replacement.

In contrast, most patients undergoing MV surgery had their MV repaired. Current literature supports the effectiveness of MV repairs, showing an association with increased survival.^27–29^ However, these patients are at higher risk for reinterventions, which was evident in our study, as those undergoing MV procedures had the highest reintervention rate. This may be attributed to the connective tissue fragility, degenerative changes, and technical challenges of maintaining durable valve function in pediatric MFS patients who are still growing, as well as the degree of myxomatous degeneration in the valve.^30^ These challenges highlight the persistent difficulty of achieving durable repairs in the context of MFS and underscore the need for further research to improve surgical strategies and outcomes in this population.

In this study, in-hospital mortality in pediatric MFS patients undergoing cardiac intervention was low at 1% (4/343) . This aligns with previous single-center studies, including those with pediatric and young adult populations, which report mortality rates between 0% and 10%.^5,11^ In the adult MFS population, increased in-hospital mortality has been primarily associated with aortic dissection and rupture. In our study, no pediatric patients were diagnosed with aortic dissection, which corresponds with the findings of Gillinov et al,^5^ where no pediatric patients had these diagnoses.

During the study period, 51/343 (15%) patients required reintervention. Single-center studies have reported a higher incidence of reoperation, ranging from 20% to 30%.^11,12,29^ This lower reintervention rate observed in our study is most likely attributable to the loss to follow-up of patients over time. It is important to emphasize that while technical surgical issues may account for some reinterventions, the higher rate observed in this population compared with other pediatric cardiac surgery patients likely reflects the progressive nature of the disease. This was evident in our study, where patients often underwent cardiac reinterventions unrelated to their initial procedure. Additionally, the higher rate of reintervention can be secondary to the higher rate of valve replacements in this population.

As previously mentioned, when a valve procedure was performed, 50% of patients required valve replacement. The type of valve used, whether mechanical or biological, was unknown. However, as children outgrow their prosthetic valves, reinterventions become inevitable and can drive the high reintervention rate observed. Cox regression analysis revealed that younger age at surgery was associated with a higher risk of reintervention. While no additional clinical data on these patients is available, this finding suggests potential benefits in delaying surgical intervention to reduce the risk of reintervention. However, delaying surgery may also expose patients to an increased risk of adverse events. Future research should focus on the impact of childhood surgical intervention on long-term outcomes in this population and on optimal timing for surgical intervention in pediatric MFS.

Long-term survival in patients with MFS has significantly improved in recent years. Previous studies have shown that median survival for patients with MFS increased from 48 years in 1972 to 61–72 years in the modern era.^31^ In our study, the long-term survival at 10 years was 94.2%. This survival rate is higher than reported in other single-center experiences, which document an overall survival rate of 79% to 88% at 10 years.^5,11^ The observed difference in survival may be partially attributed to loss of follow-up as patients transition from pediatric to adult care facilities and possible mortality events out of a hospital or a non-PHIS hospital. Consequently, the survival rates presented in this study likely represent a best-case scenario and should be interpreted with caution. Nonetheless, these findings highlight the critical need to improve patient tracking and follow-up during care transitions to better understand outcomes and prognosis in uncommon patient groups, such as pediatric MFS.

## Limitations

The limitations of the current study should be noted. First, its retrospective nature, coupled with the utilization of data extracted from a large administrative database inherently restricts the ability to establish causative relationships and lacks significant clinical detail. Second, ICD procedural codes do not allow the determination of the specific procedure performed, as such specific techniques utilized are unknown. Furthermore, PHIS includes only encounters in pediatric hospitals. As patients reach adulthood it is possible that their care is transitioned to an adult facility, where such data in these encounters will not be included. Therefore, long-term outcomes and reinterventions presented in this work represent the best-case scenario with the risk of underreporting reinterventions and mortality. Despite these limitations, to our knowledge this work represents the first multiinstitutional analysis reporting on the incidence and outcomes of pediatric MFS patients undergoing surgical interventions.

## Conclusion

This study represents to our knowledge the largest sample to date in pediatric MFS patients undergoing cardiovascular surgical intervention. Operations for cardiovascular complications of MFS can be performed safely in appropriately selected children with low morbidity and mortality and with good short-term and long-term outcomes. These findings support the safety and efficacy of surgical intervention in this population. Further work on long-term morbidity and mortality is needed to better elucidate outcomes of surgical intervention during childhood.

## Supplemental Material

sj-docx-1-pch-10.1177_21501351251335474 - Supplemental material for Surgical Intervention in Pediatric Marfan Syndrome: A Multiinstitutional Study Supplemental material, sj-docx-1-pch-10.1177_21501351251335474 for Surgical Intervention in Pediatric Marfan Syndrome: A Multiinstitutional Study by Mario O’Connor, Andrew Well, Maria E. Hoyos, Catherine E. Stauber, Charles D. Fraser and Hugo Martinez in World Journal for Pediatric and Congenital Heart Surgery
