# Supplementary material for: Surgical Intervention in Pediatric Marfan Syndrome: A Multiinstitutional Study
Source: World J Pediatr Congenit Heart Surg. 2025 May 15;16(6):782–9. doi: 10.1177/21501351251335474 (PMC12504790; doi:10.1177/21501351251335474)

Supplemental Material

Supplemental Table 1. Valve Procedures

| Procedure | Overall | Aortic / Aortic + AV / AV Procedure  n = 141 | Aortic + MV / AV + MV  n = 40 | MV Procedure  n = 62 |
| --- | --- | --- | --- | --- |
| Replacement | 129 (50) | 89 (63) | AV: 11 (30)  MV: 5 (13) | 24 (39) |
| Repair | 125 (48) | 46 (33) | AV: 6 (15)  MV: 35 (88) | 38 (61) |
| Ross Procedure | 6 (2) | 6 (4) | - | - |

Supplemental Table 2. Multivariable Cox Regression Analysis for Any Cardiac Reintervention

| Variable | HR | 95% CI | p-value |
| --- | --- | --- | --- |
| Age (Median) in Years [IQR] | 0.94 | 0.89 – 0.99 | **0.023** |
| AV procedure | 1.11 | 0.61 – 2.01 | 0.713 |
| MV procedure | 0.83 | 0.38 – 1.80 | 0.649 |
| Aortic procedure | 0.94 | 0.52 – 1.67 | 0.835 |

AV: Aortic valve; MV: Mitral valve

Supplemental Figure 1


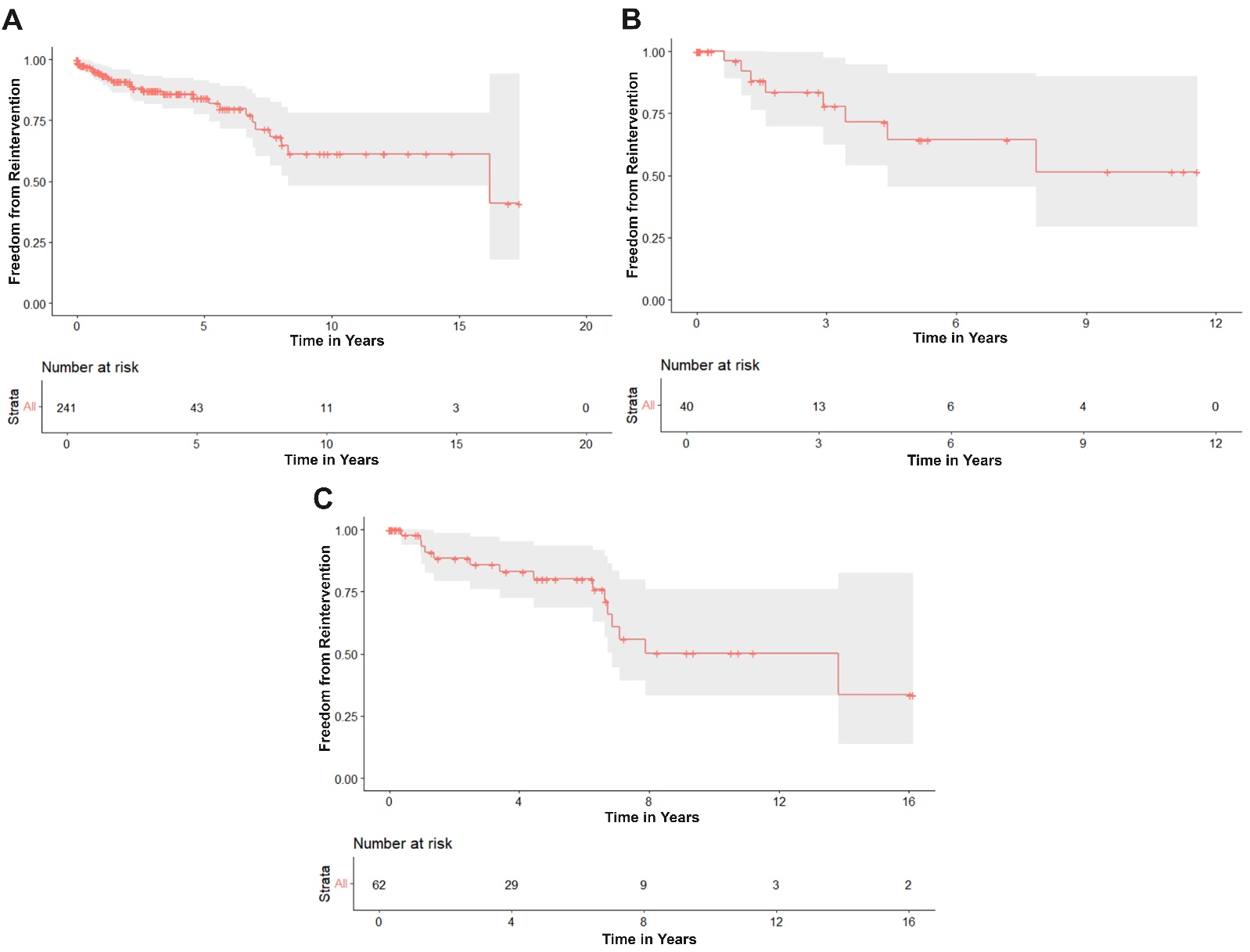

Supplement: sj-docx-1-pch-10.1177_21501351251335474 - Supplemental material for Surgical Intervention in Pediatric Marfan Syndrome: A Multiinstitutional Study [file sj-docx-1-pch-10.1177_21501351251335474.docx]
